# Diagnoses of Mental Health Disorders Among Active Component U.S. Armed Forces, 2019–2023

**Published:** 2024-12-20

**Authors:** Armed Forces Health Surveillance Division Defense Health Agency, US

## Abstract

Mental health disorders have historically accounted for significant morbidity, health care provision, disability, and attrition from military service. From 2019 through 2023, a total of 541,672 active component service members of the U.S. Armed Forces were diagnosed with at least 1 mental health disorder. Crude annual incidence rates of at least 1 mental health disorder decreased from 2019 to 2020, but then increased continually from 2021 until 2023. Most incident mental health disorder diagnoses during the study period were attributable to adjustment disorders, anxiety disorders, depressive disorders, ‘other’ mental health disorders, and alcohol-related disorders. Efforts to assist and treat service members should continue to promote help-seeking behavior to improve their psychological and emotional well-being and reduce the burden of mental health disorders, especially as rates have been increasing since the COVID-19 pandemic.

**What are the new findings?:**

Annual incidence rates for service members diagnosed with at least 1 mental health disorder increased from 2021 through 2023, coincident with the COVID-19 pandemic. Incidence rates for anxiety disorder and post-traumatic stress disorder increased substantially from 2019 to 2023, nearly doubling during that period.

**What is the impact on readiness and force health protection?:**

As service members continue to experience increased rates of mental health disorders after the COVID-19 pandemic, help-seeking behaviors to address psychological as well as emotional well-being should be prioritized to maintain force readiness.

## 1 BACKGROUND

In 2023, mental health disorders accounted for the largest total number of hospital bed days and the second highest total number of medical encounters for members of the active component of the U.S. Armed Forces.^1^ The most recent *MSMR* update on mental health disorders, from 2016 through 2020, found relatively stable incidence rates for all conditions evaluated, with the exception of adjustment disorders and depressive disorders.^2^ In preceding periods, incident diagnoses of mental health disorders among active component service members (ACSMs) increased, by 65% from 2000 to 2011, largely attributable to diagnoses for adjustment disorders, depression, anxiety, and post-traumatic stress disorder (PTSD).^3^ In general, crude incidence rates of mental health disorders have been observed to be highest among service members in the Army, females, and in younger age groups.^2,3,4^

This report summarizes the numbers, types, and rates of incident mental health disorder diagnoses among U.S. ACSMs over a 5-year surveillance period, from 2019 through 2023. This update separates 3 additional mental health disorders (acute stress disorder, eating disorders, and factitious disorders) that were previously combined in the ‘other mental health disorders’ category in prior *MSMR* articles. Additionally, data on the ‘mental health problems’ categories, which has been renamed ‘nonmedical factors influencing health’, are no longer provided in this report, but will be reported in a separate *MSMR* article.^5^

## 2 METHODS

The surveillance period for this report ranged from January 1, 2019 through December 31, 2023. The surveillance population included all individuals who served in the active components of the U.S. Army, Navy, Air Force, Marine Corps, Coast Guard, and Space Force at any time during the surveillance period. Due to Space Force personnel data only available since 2023, Space Force members were combined with Air Force personnel for this analysis.

All data used to determine mental health diagnoses were derived from records routinely maintained in the Defense Medical Surveillance System (DMSS). These records document both ambulatory encounters and hospitalizations of active component members of the U.S. Armed Forces in fixed military and civilian (if reimbursed through the Military Health System [MHS]) hospitals and clinics. Diagnoses were also derived from records of medical encounters of deployed service members documented in the Theater Medical Data Store (TMDS) in DMSS.

For surveillance purposes, mental health disorders were ascertained from records of medical encounters that included mental health disorder-specific diagnoses (ICD-9: 290-319; ICD-10: F01-F99) (**Table 1**) in the first or second diagnostic position. Although the MHS transitioned to ICD-10 coding on October 1, 2015, ICD-9 codes were included in this analysis because some TMDS encounters still contain ICD-9 diagnoses, and the ICD-9 diagnoses were needed to identify and exclude prevalent cases documented in records preceding October 1, 2015. Diagnoses of pervasive developmental disorder (ICD-9: 299.*; ICD-10: F84.*), specific delays in development (ICD-9: 315.*; ICD-10: F80.*–F82.*, F88–F89), mental retardation (ICD-9: 317.*–319.*; ICD-10: F70–F79), tobacco use disorder and nicotine dependence (ICD-9: 305.1; ICD-10: F17.*), and post-concussion syndrome (ICD-9: 310.2; ICD-10: F07.81) were excluded from the analysis.

Each incident diagnosis of a mental health disorder was defined using the corresponding Armed Forces Health Surveillance Case Definition.^5^ For most mental health disorders, a case was defined by a hospitalization with an indicator diagnosis in the first or second diagnostic position; or 2 outpatient or TMDS visits within 180 days documented with indicator diagnoses (from the same mental health disorder category) in the first or second diagnostic position; or a single outpatient visit in a psychiatric or mental health care specialty setting (defined by Medical Expense and Performance Reporting System [MEPRS] code beginning with ‘BF’) with an indicator diagnosis in the first or second diagnostic position.

The surveillance case definitions for schizophrenia, acute stress disorder, and eating disorders included some exceptions to the case parameters described. The case definition for schizophrenia required either a single hospitalization with a diagnosis of schizophrenia in the first or second diagnostic position, or 4 outpatient or TMDS encounters with a diagnosis of schizophrenia in the first or second diagnostic position. Schizophrenia cases who remained in the military for more than 2 years after becoming an incident case were excluded, as those cases were assumed to have been misdiagnosed. The case definition for the acute stress disorders required 1 encounter with an indicator diagnosis in any diagnostic position, due to its transient diagnosis. Eating disorder cases required 1 inpatient encounter with an indicator diagnosis in the first or second diagnostic position, or a single outpatient or TMDS encounter with an indicator diagnosis in the primary diagnostic position.

Service members who were diagnosed with 1 or more mental health disorders before the surveillance period (i.e., prevalent cases) were not considered at risk of incident diagnoses of the same conditions during the surveillance period. Service members who were diagnosed with more than 1 mental health disorder during the surveillance period were considered incident cases in each category in which they fulfilled case-defining criteria. Service members could be incident cases only once in each specific mental health disorder category.

## 3 RESULTS

During the 5-year surveillance period, 541,672 ACSMs were diagnosed with at least 1 mental health disorder; of those individuals, 255,108 (47.1%) were diagnosed with mental health disorders in more than 1 diagnostic category (**Table 2**). Overall, 966,227 incident diagnoses of mental health disorders were recorded in all diagnostic categories. Annual numbers and rates of incident diagnoses of at least 1 mental health disorder decreased from 8,795 cases per 100,000 person-years (p-yrs) in 2019 to 8,391 cases per 100,000 p-yrs in 2020, and then increased from 2021 to 2023, with a peak incidence rate of 11,706 cases per 100,000 p-yrs in 2023 (**Table 2**).

Over the entire period, 94.8% of all incident mental health disorder diagnoses were attributable to adjustment disorders (n=282,960, 29.3%), anxiety disorders (n=187,949, 19.5%), depressive disorders (n=168,519, 17.4%), ‘other’ mental health disorders (n=119,536, 12.4%); PTSD (n=86,216, 8.9%), and alcohol-related disorders (n=70,729, 7.3%) (**Table 2**). In comparison, relatively few incident diagnoses were attributable to substance-related disorders (n=15,901, 1.6%), personality disorders (n=15,833, 1.6%), bipolar disorder (n=8,454, 0.9%), other psychoses (n=3,917, 0.4%), eating disorders (n=3,380, 0.3%), schizophrenia (n=1,506, 0.2%), acute stress disorders (n=1,220, 0.1%), and factitious disorders (n=107, 0.01%).

It was common for individuals with any mental health disorder to also experience an adjustment disorder diagnosis during the surveillance period. This co-occurrence ranged from 37.1% of substance-related disorder cases to 61.8% of personality disorder cases (**Table 3**). Depressive disorders were also commonly diagnosed with all other mental health disorders, ranging from 26.9% of those with a substance-related disorder to 60.1% of those with a bipolar disorder. Incident cases of anxiety disorders were also frequently diagnosed among cases of bipolar disorder (46.1%), factitious disorders (43.9%), eating disorders (43.6%), depressive disorders (43.2%), personality disorders (40.2%), PTSD (40.1%), and schizophrenia (36.5%).

Crude annual rates of incident diagnoses of adjustment disorders, alcohol-related disorders, substance-related disorders, personality disorders, schizophrenia, other psychoses, acute stress disorders, eating disorders, and other mental health disorders followed a general pattern of decreasing or stabilizing from 2019 to 2020, increasing in 2021 and 2022, and then decreasing or stabilizing in 2023 (**Table 2**). Over the 5-year surveillance period, the largest increase in annual incidence of mental health disorders was observed for anxiety disorders (89.8%) and PTSD (86.4%). Rates of bipolar disorders increased from 2019 to 2022 and then decreased slightly in 2023.

In general, overall rates of most incident mental health disorder diagnoses were higher among female service members, with exceptions for schizophrenia, for which rates were similar for both sexes, and alcohol- and substance-related disorders, for which rates were higher among male service members (**Figure 1a**,**Figure 1b**,**Figure 2a**,**Figure 2b**). Rates of most mental health disorder diagnoses declined with increasing age, from the 20-24-year age group and older (**Figure 3**). Adjustment disorder was the only condition for which the crude overall incidence rate was higher among the youngest (less than 20 years old) service members, compared to all other age groups. Rates of alcohol- and substance-related disorders, bipolar disorders, personality disorders, schizophrenia, and eating disorders were highest among service members aged 20-24 years (**Figure 3**). In contrast, the rates of PTSD increased with age, ‘other’ mental health disorders decreased with age, while crude incidence rates of anxiety disorders and depressive disorders fluctuated throughout the age groups.

Overall incidence rates of mental health disorders were highest in the Army, although the Navy accounted for the highest rates of depressive disorders, bipolar disorder, and personality disorders, while the Coast Guard accounted for the highest rates of acute stress disorders (**Figure 4**). Crude overall incidence rates of most mental health disorders were highest among ACSMs in health care occupations, although crude incidence rates of alcohol-related disorders, substance-related disorders, and factitious disorders were highest among those in combat-related occupations (**Figure 5**). Service members in the motor transport occupations evinced the highest crude incidence rates of other psychoses and schizophrenia.

**Figure 6:**
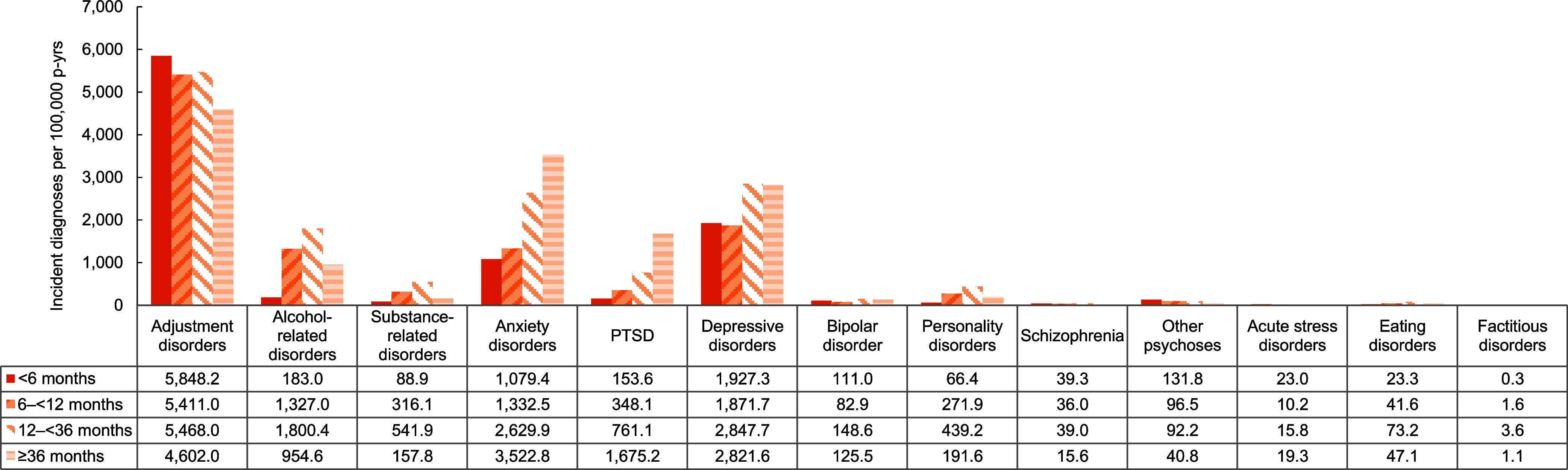
Incidence Rates of Mental Health Disorder Diagnoses, by Time in Service, Active Component, U.S. Armed Forces, 2019–2023

Rates of mental health disorder diagnoses increased by time in service until 36 months for most disorders, with rates of anxiety disorders and PTSD increasing after 36 months of service (**Figure 6**). Rates of adjustment disorders, schizophrenia, other psychoses, and acute stress disorders were highest during the first 6 months of military service, however. Finally, overall rates of incident anxiety disorders, PTSD, acute stress disorders, and ‘other’ mental health disorders were higher among service members who had ever deployed to a U.S. Central Command (CENTCOM) area of responsibility (AOR) (data not shown).

## 4 DISCUSSION

This report provides an update on incident diagnoses for mental health disorders among ACSMs of the U.S. Armed Forces from 2019 through 2023. These trends demonstrate a growing need for mental health services among U.S. military members, as the incidence rate of any mental health diagnosis increased by almost 40% between 2019 and 2023. Disorders related to adjustment, anxiety, and depression remain the most common mental health diagnoses, as documented in previous *MSMR* reports.^2,3^ Notably, incidence rates for anxiety disorders and PTSD increased substantially, almost doubling from 2019 to 2023.

A temporary decline in the incidence of most mental health disorders was observed between 2019 and 2020, corresponding with the beginning of the coronavirus disease (COVID-19) pandemic. This decreasing trend does not reflect reports from the Centers for Disease Control and Prevention (CDC), which documented an increase in adverse mental health conditions associated with effects of the COVID-19 pandemic.^6,7^ This decrease may, instead, be related to service members choosing to defer care due to the pandemic, similar to temporary disruptions in routine and non-emergency medical care observed in the general U.S. population.^8^ Consequently, the temporary decline observed in this study may be related to changes to access and provision of mental health care services during the pandemic.

From 2019 to 2022, the percentage of general U.S. adults with anxiety (from 15.6% to 18.2%) and depression (from 18.5% to 21.4%) symptoms increased significantly.^9^ Subsequent increases in anxiety and depressive disorders following the COVID-19 pandemic were also observed among male and female ACSMs. Prior *MSMR* reports indicate that approximately one-third of anxiety disorder cases between 2000 to 2011 had co-occurring diagnoses of either adjustment or depressive disorder.^10^ Co-occurring diagnoses persist in the current report, which documents both adjustment disorders (43.5%) and depressive disorders (38.7%) as the leading 2 co-occurring diagnoses from 2019 to 2023 for ACSMs with incident anxiety disorder diagnoses. Comparable to *MSMR* reports from the last 2 decades, incidence rates of anxiety disorders remain highest among female service members and health care occupations.^3^

The rate of PTSD among ACSMs increased nearly six-fold from 2003 to 2008, likely reflecting the psychological effects among participants in Operations Iraqi Freedom and Enduring Freedom.^3^ While this report also documents a subsequent peacetime operation increase in PTSD rates, the demographic distributions differ from prior reports. From 2000 to 2011, incidence rates of PTSD were higher among men and decreased with age.^3^ In contrast, from 2019 to 2023 the incidence of PTSD in female ACSMs was consistently twice the rate of male counterparts, while also increasing with age. These findings likely reflect the changing demographics of the force, now representing increasing numbers of women,^11^ and may also be related to sex-specific differences in comorbid mental health disorders that can predispose ACSMs to higher PTSD rates.^12^ Congruent with prior reports, service members in health care occupations continued to represent high rates of PTSD, potentially reflecting the psychological stresses inherent to many health care roles in both peace and wartime operations.

The 2018 Health Risk Behavior Survey (HRBS) indicates that approximately 7% of service members reported needing—but not receiving—mental health services in the past 12 months. Furthermore, over one-third of all active component HRBS respondents suggested that seeking mental health services damages one’s military career.^13^ These findings underscore the limitations of interpreting these results, which are based on standardized administrative records and may not be reliable indicators for the true burden of mental health disorders among military service members. This report may underestimate mental health disorder incidence if service members do not seek care or receive care not routinely documented as ICD-9/10-coded diagnoses (e.g., private practitioner, counseling or advocacy support center, chaplains); if mental health disorders were not diagnosed nor reported on standardized records of care; or if diagnoses were miscoded or incorrectly transcribed on the centrally transmitted records. Conversely, some conditions may have been erroneously diagnosed or miscoded as mental health disorders (e.g., screening visits), which may contribute to an over-estimation of the true burden of disease. This report documents recent changes to the case surveillance definitions for mental health disorders, maintained by the Armed Forces Health Surveillance Division.

This update presents results for 3 new case categories, including acute stress disorder, eating disorders, and factitious disorders; diagnoses under these categories were previously combined in the ‘other mental health disorders’ categories presented in prior *MSMR* articles.^2,3,4^ Additionally, prior reports present data for a generalized “mental health problems” category, which included Z-code diagnosis codes related to factors influencing the health status of an individual warranting clinical attention. While those ‘Z’ codes are no longer presented in this report, a separate report will summarize a new case classification for the Z codes related to mental health disorders as “non-medical factors influencing health.” The estimates of the numbers, natures, and rates of illnesses and injuries of surveillance interest depend on specifications of the surveillance case definitions; thus, changes to case definitions should be considered when comparing this report to prior data. In addition, the analyses reported herein summarize the experiences of individuals while serving in an active component of the U.S. military and do not include mental health disorders and mental health problems that affected members of reserve components or veterans of recent military service who received care outside the MHS.

In 2023, mental health disorders accounted for more hospital bed days than any other morbidity-related diagnostic category, contributing to over half (54.8%) of all hospital bed days among ACSMs.^1^ A substantial proportion of those bed days occurred in non-military medical facilities. Policy implications from the published HRBS call for additional research to identify the reasons service members seek mental health care outside the MHS.

In September 2024, the Department of Defense revised Instruction 6490.08 and established a Department policy to promote health-seeking behaviors for mental health services. This policy emphasizes unrestricted, non-stigmatizing access to mental health care services, including voluntary substance misuse education, as essential for maintaining the health and readiness of the total force.^14^ As the burden of mental health disorders continues to increase during a period of policy change, ongoing surveillance and further analyses are warranted to better understand the true burden of disease, along with related health care access and use. The results from this report underscore the need for mental health services to address a range of mental health comorbidities within the active component of the U.S. Armed Forces.

## Figures and Tables

**Table 1:** Mental Health Categories and ICD-9 / ICD-10 Diagnostic Codes

Diagnostic Category	ICD–9 Codes	ICD–10 Codes
Mental health disorders		
Acute stress disorders	308.*	F43.0
Adjustment disorders	309.** (excluding 309.81)	F43.2, F43.2*, F43.8, F43.9, F93.0, F94.8, F94.9
Alcohol-related disorders	291.0, 291.81, 303.9, 303.9*, 303.00, 303.0*, 305.00, 305.0*	F10.1*, F10.2*
Substance-related disorders	304.*, 305.2*–305.9*	F11.2*, F12.2*, F13.2*, F14.2*, F15.2*, F16.2*, F18.2*, F19.2*, F11.1*, F12.1* F13.1*, F14.1*, F15.1*, F16.1*, F18.1*, F19.1*
Anxiety disorders	300.0*, 300.2*, 300.3	F40.*, F41.*, F42.*
PTSD	309.81	F43.1, F43.10–F43.12
Depressive disorders	296.2*, 296.3*, 296.82, 296.9*, 300.4, 311	F32.*, F33.*, F34, F34.1, F34.8, F34.9, F39, F348.1, F34.89
Eating disorders	307.1, 307.51, 307.59, 307.50	F500.*, F502, F508.*, F509
Factitious disorders	301.51, 300.16, 300.19	F681.*
Bipolar disorder	296.0*, 296.1*, 296.4*, 296.5*, 296.6*, 296.7, 296.8* (except 296.82), 301.13	F30.*, F31.*, F34.0
Personality disorders	301.** (excluding 301.13, 301.50, 301.52)	F21, F60.*
Schizophrenia	295**	F20*, F25*
Other psychoses	293.81, 293.82, 297.0*, 298.0*	F06.0, F06.2, F22–F24, F28, F29
Other mental health disorders	Any other code between 290–319 (excluding 299.*, 305.1, 310.2, 315.*, 317.*–319.*)	Any other code between F01–F99 (excluding F07.81, F70–F79, F17.*, F80.*–F82.*, F84.*, F88–F89)

Abbreviations: ICD-9/ICD-10, International Classification of Diseases, 9th and 10th revisions; PTSD, post-traumatic stress disorder.

*Asterisk indicates that any subsequent digit or character is included.

**Table 2:** Incident Diagnoses and Rates of Mental Health Disorders, Active Component, U.S. Armed Forces, 2019–2023

Total, 2019-2023			2019		2020		2021		2022		2023	
Category^a^	No.	Rate^b^	No.	Rate^b^	No.	Rate^b^	No.	Rate^b^	No.	Rate^b^
Diagnoses										
Adjustment disorders	282,960	4,915	52,798	4,485	50,213	4,259	61,437	5,224	62,286	5,518	56,226	5,128
Alcohol-related disorders	70,729	1,105	14,800	1,150	13,182	1,016	14,285	1,095	14,869	1,173	13,593	1,096
Substance-related disorders	15,901	240	3,170	238	2,955	220	3,369	249	3,485	265	2,922	227
Anxiety disorders	187,949	3,071	27,672	2,218	28,055	2,239	37,845	3,022	45,437	3,774	48,940	4,210
PTSD	86,216	1,334	12,584	964	13,016	990	17,383	1,318	20,847	1,631	22,386	1,797
Depressive disorders	168,519	2,726	28,481	2,271	26,745	2,121	34,554	2,735	39,156	3,211	39,583	3,341
Bipolar disorder	8,454	127	1,366	102	1,486	110	1,834	136	1,959	149	1,809	140
Personality disorders	15,833	239	2,998	225	2,765	206	3,303	245	3,584	273	3,183	248
Schizophrenia	1,506	23	288	22	261	19	311	23	327	25	319	25
Other psychoses	3,917	59	759	57	732	54	844	62	816	62	766	59
Acute stress disorders	1,220	18	236	18	199	15	263	19	290	22	232	18
Eating disorders	3,380	51	509	38	497	37	709	52	882	67	783	61
Factitious disorders	107	2	32	2	17	1	16	1	24	2	18	1
Other mental health disorders	119,536	1,930	22,413	1,794	20,071	1,594	24,438	1,932	26,740	2,182	25,874	2,167
Total	966,227		168,106		160,194		200,591		220,702		216,634	
Individuals										
<1 type of mental health diagnosis^c^	255,108	3,834	35,648	2,665	33,625	2,494	43,663	3,219	48,858	3,702	47,934	3,710
Any mental health diagnosis^d^	541,672	8,141	117,654	8,795	113,116	8,391	139,611	10,292	152,817	11,580	151,228	11,706

Abbreviations: No., number; PTSD, post-traumatic stress disorder.

^a^An individual may be a case within a category only once per lifetime (censored person-time).

^b^Rate per 100,000 person-years.

^c^Rate per 100,000 person-years (individual continually at risk, uncensored person-time).

^d^Defined as a unique occurrence of any mental health diagnosis.

**Table 3:** Comorbid Incident Mental Health Disorder Diagnoses,^a^ Active Component, U.S. Armed Forces, 2019–2023

	Adjustment Disorders		Alcohol-related Disorder		Substance-related Disorder		Anxiety		PTSD		Depression		Bipolar Disorder		Personality Disorders		Schizophrenia		Other Psychoses		Acute Stress Disorder		Eating Disorders		Factitious Disorder		Other	
	No.	%	No.	%	No.	%	No.	%	No.	%	No.	%	No.	%	No.	%	No.	%	No.	%	No.	%	No.	%
Adjustment disorders	282,960	-	27,472	38.8	5,904	37.1	81,779	43.5	39,929	46.3	84,920	50.4	3,933	46.5	9,786	61.8	640	42.5	1,892	48.3	507	41.6	1,569	46.4	56	52.3	43,941	36.8
Alcohol-related disorder	27,472	9.7	70,729	-	7,533	47.4	16,888	9.0	9,380	10.9	21,739	12.9	1,518	18.0	3,221	20.3	268	17.8	736	18.8	105	8.6	426	12.6	10	9.4	17,256	14.4
Substance-related disorder	5,904	2.1	7,533	10.7	15,901	-	3,225	1.7	1,530	1.8	4,269	2.5	555	6.6	782	4.9	160	10.6	474	12.1	27	2.2	62	1.8	2	1.9	5,878	4.9
Anxiety disorders	81,779	28.9	16,888	23.9	3,225	20.3	187,949	-	34,607	40.1	72,801	43.2	3,901	46.1	6,369	40.2	549	36.5	1,363	34.8	416	34.1	1,475	43.6	47	43.9	32,815	27.5
PTSD	39,929	14.1	9,380	13.3	1,530	9.6	34,607	18.4	86,216	-	34,623	20.6	2,464	29.2	3,715	23.5	245	16.3	681	17.4	285	23.4	886	26.2	18	16.8	16,900	14.1
Depressive disorders	84,920	30.0	21,739	30.7	4,269	26.9	72,801	38.7	34,623	40.2	168,519	-	5,079	60.1	8,713	55.0	799	53.1	1,965	50.2	384	31.5	1,624	48.1	48	44.9	30,633	25.6
Bipolar disorder	3,933	1.4	1,518	2.2	555	3.5	3,901	2.1	2,464	2.9	5,079	3.0	8,454	-	1,272	8.0	306	20.3	759	19.4	39	3.2	163	4.8	7	6.5	1,968	1.7
Personality disorders	9,786	3.5	3,221	4.6	782	4.9	6,369	3.4	3,715	4.3	8,713	5.2	1,272	15.1	15,833	-	217	14.4	612	15.6	48	3.9	357	10.6	23	21.5	3,991	3.3
Schizophrenia	640	0.2	268	0.4	160	1.0	549	0.3	245	0.3	799	0.5	306	3.6	217	1.4	1,506	-	981	25.0	7	0.6	17	0.5	3	2.8	411	0.3
Other psychoses	1,892	0.7	736	1.0	474	3.0	1,363	0.7	681	0.8	1,965	1.2	759	9.0	612	3.9	981	65.1	3,917	-	18	1.5	32	1.0	13	12.2	1,170	1.0
Acute stress disorders	507	0.2	105	0.2	27	0.2	416	0.2	285	0.3	384	0.2	39	0.5	48	0.3	7	0.5	18	0.5	1,220	-	9	0.3	0	0.0	342	0.3
Eating disorders	1,569	0.6	426	0.6	62	0.4	1,475	0.8	886	1.0	1,624	1.0	163	1.9	357	2.3	17	1.1	32	0.8	9	0.7	3,380	-	1	0.9	1,198	1.0
Factitious disorders	56	0.0	10	0.0	2	0.0	47	0.0	18	0.0	48	0.0	7	0.1	23	0.2	3	0.2	13	0.3	0	0.0	1	0.0	107	-	41	0.0
Total	282,960		70,729		15,901		187,949		86,216		168,519		8,454		15,833		1,506		3,917		1,220		3,380		107		119,536	

Abbreviations: No., number; PTSD, post-traumatic stress disorder.

^a^Mental health disorder diagnoses at any time during the surveillance period.

**Figure 1a:**
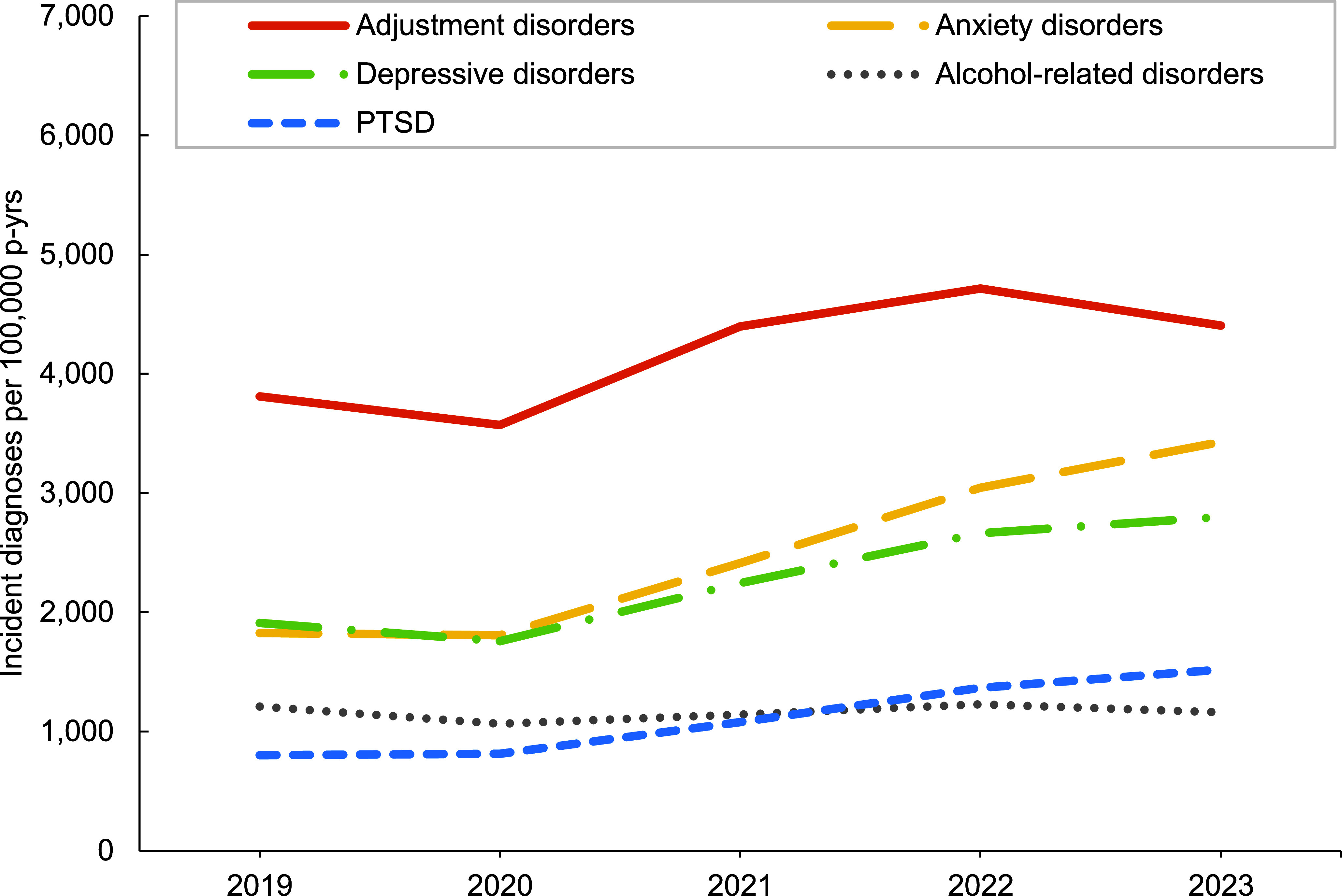
Annual Incidence Rates, Leading 5 Mental Health Disorder Diagnoses Among Male Active Component Service Members, U.S. Armed Forces, 2019–2023

**Figure 1b:**
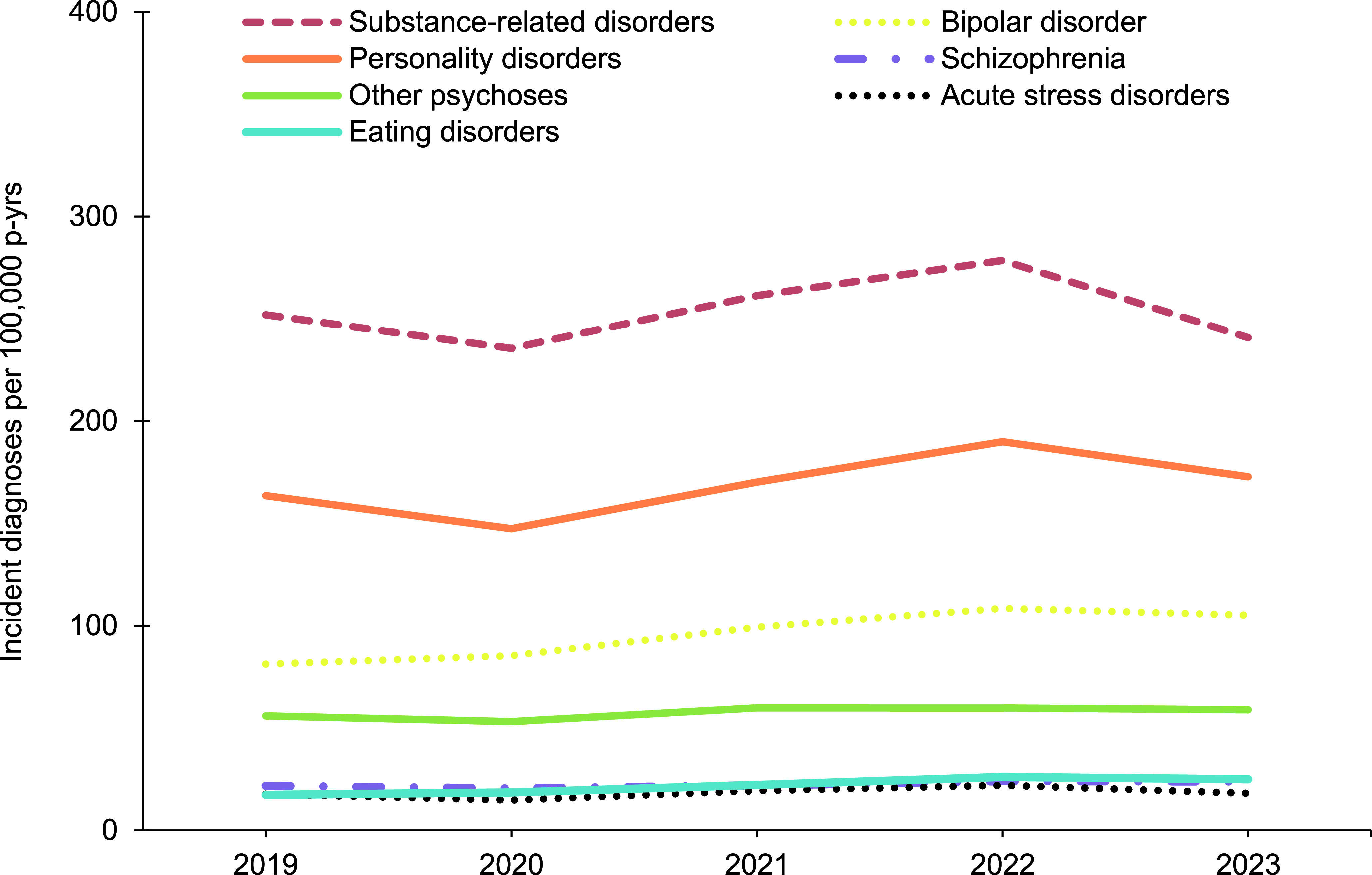
Annual Incidence Rates^a^, Mental Health Diagnoses Following the Leading 5 Disorders, Male Active Component Service Members, U.S. Armed Forces, 2019–2023

**Figure 2a:**
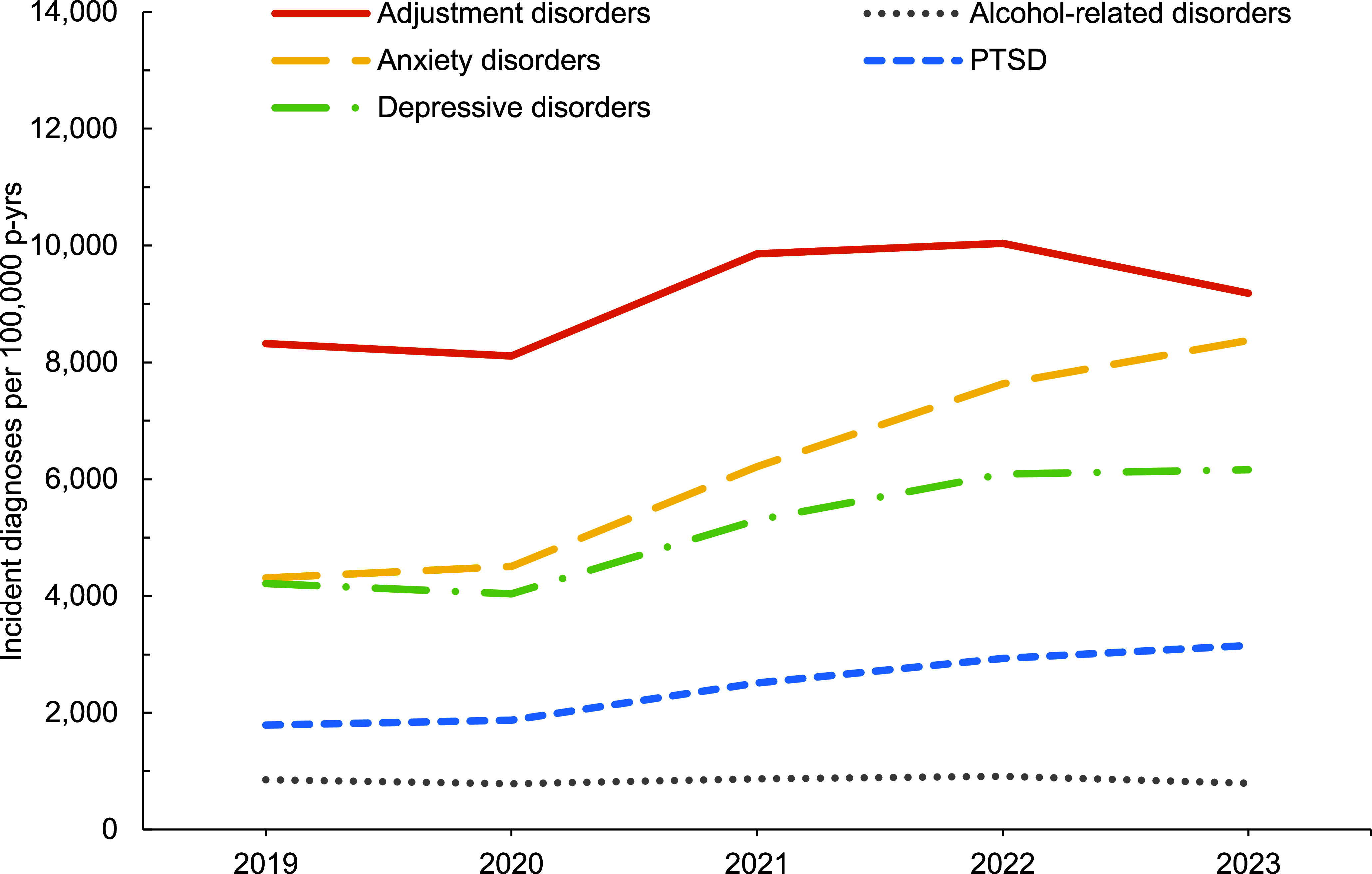
Annual Incidence Rates, Leading 5 Mental Health Disorder Diagnoses Among Female Active Component Service Members, U.S. Armed Forces, 2019–2024

**Figure 2b:**
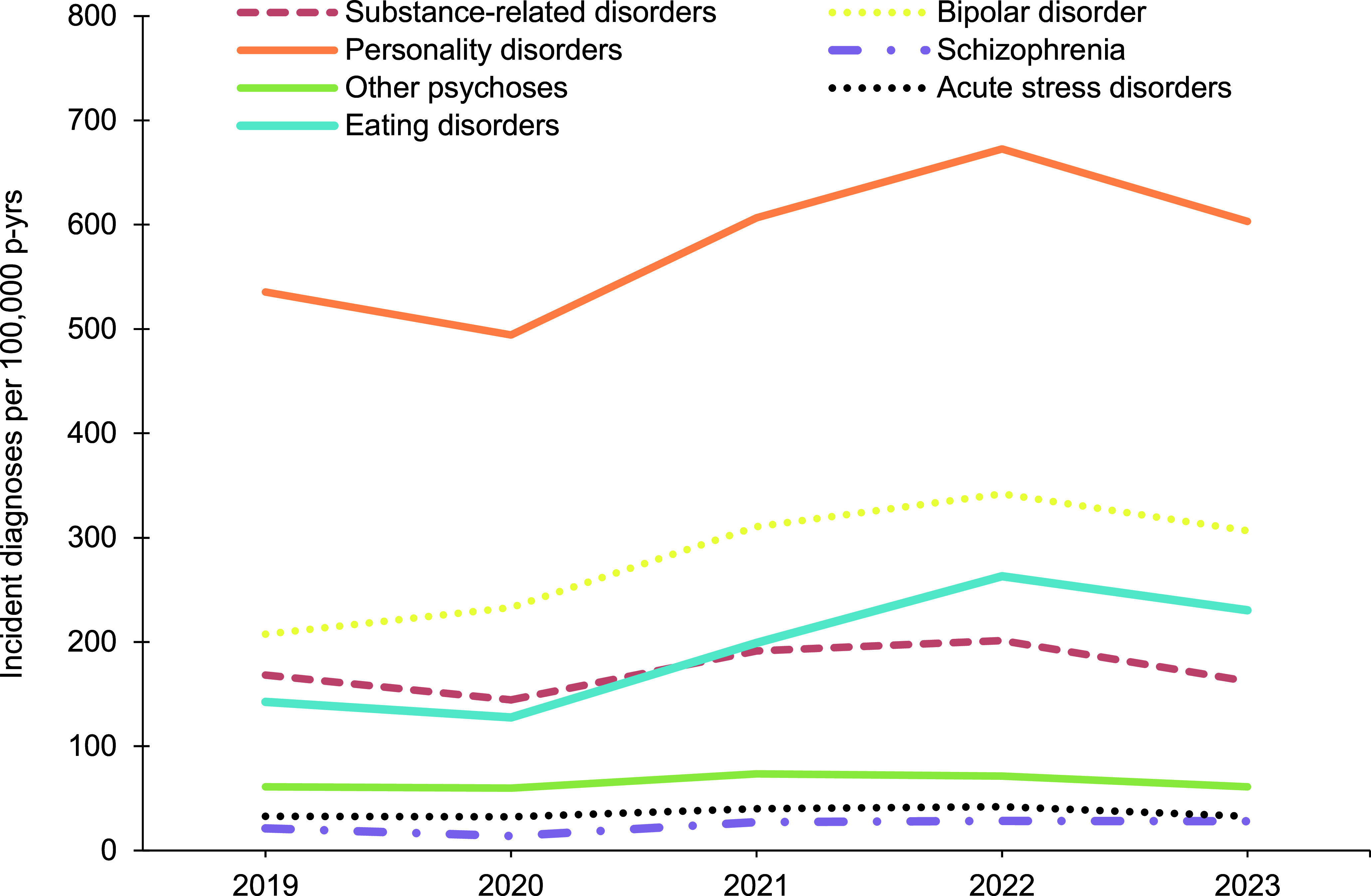
Annual Incidence Rates^a^, Mental Health Diagnoses Following the Leading 5 Disorders, Female Active Component Service Members, U.S. Armed Forces, 2019–2023

**Figure 3:**
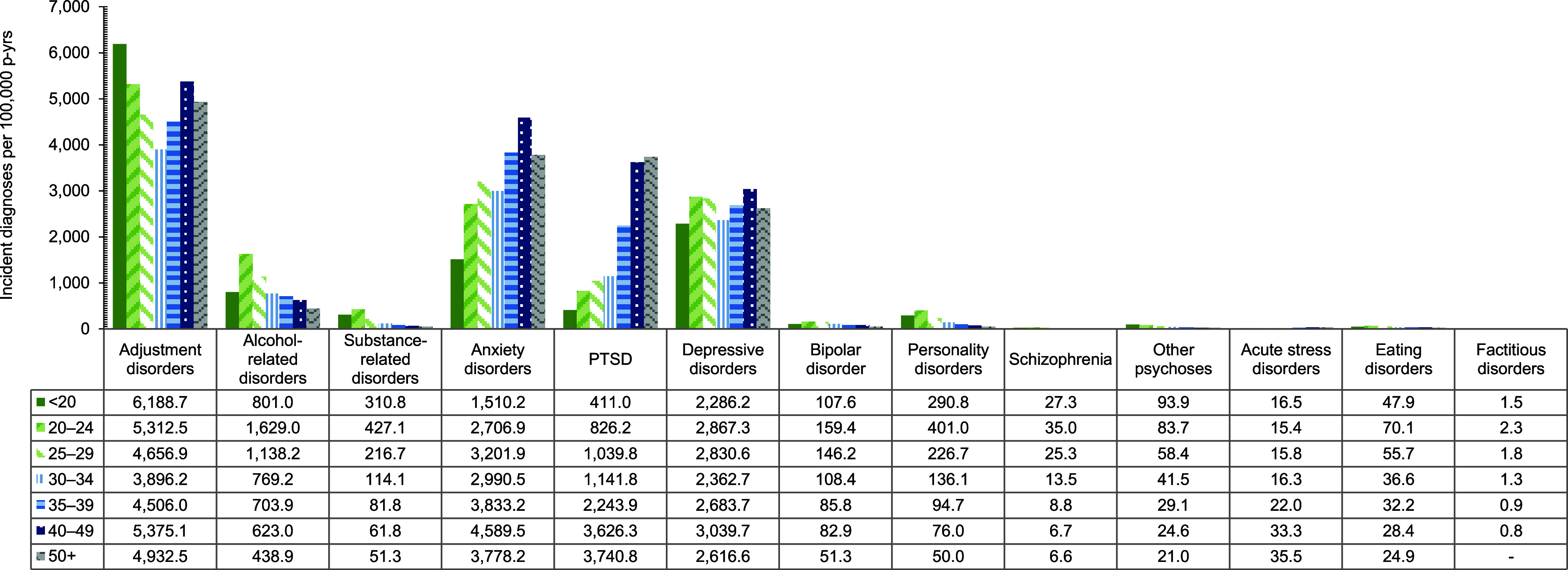
Incidence Rates of Mental Health Disorder Diagnoses, by Category and Age Group, Active Component, U.S. Armed Forces, 2019–2023

**Figure 4:**
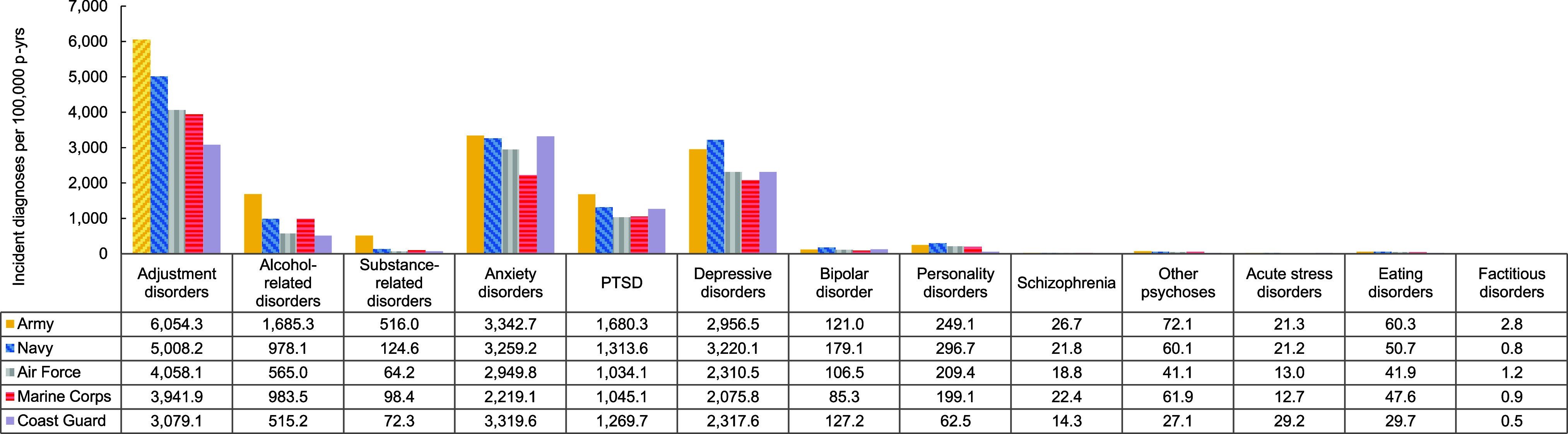
Incidence Rates of Mental Health Disorder Diagnoses, by Category and Service, Active Component, U.S. Armed Forces, 2019–2023

**Figure 5:**
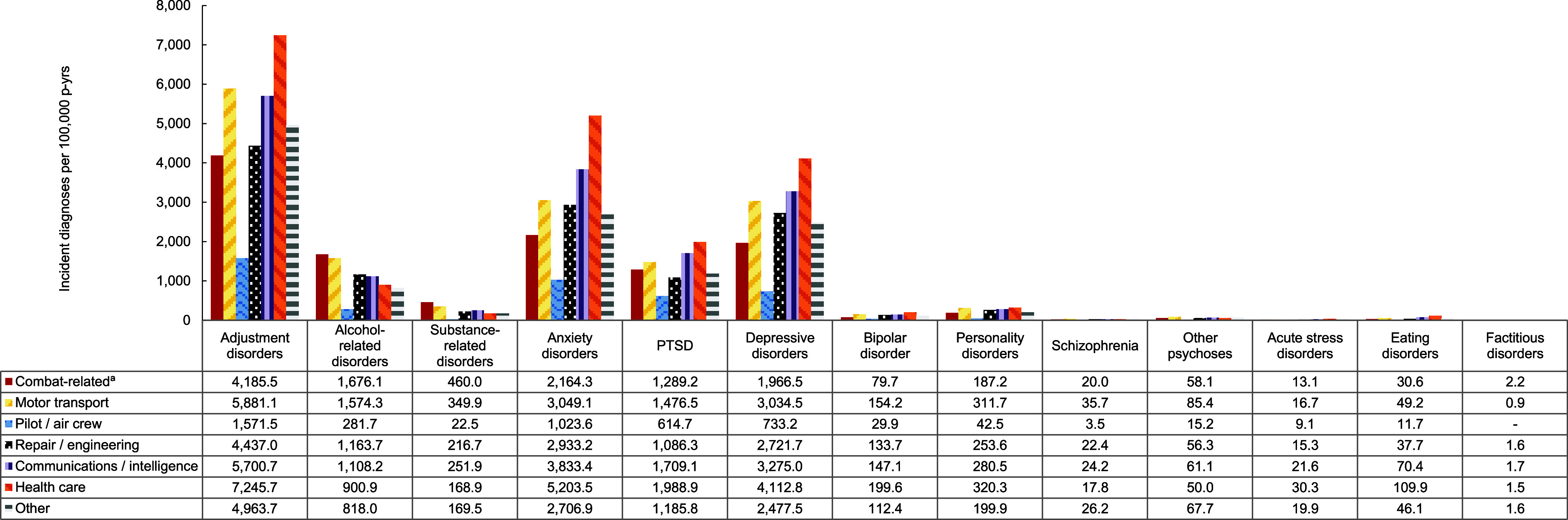
Incidence Rates of Mental Health Disorder Diagnoses, by Category and Military Occupation, Active Component, U.S. Armed Forces, 2019–2023

**Figure 3 Supplement:**
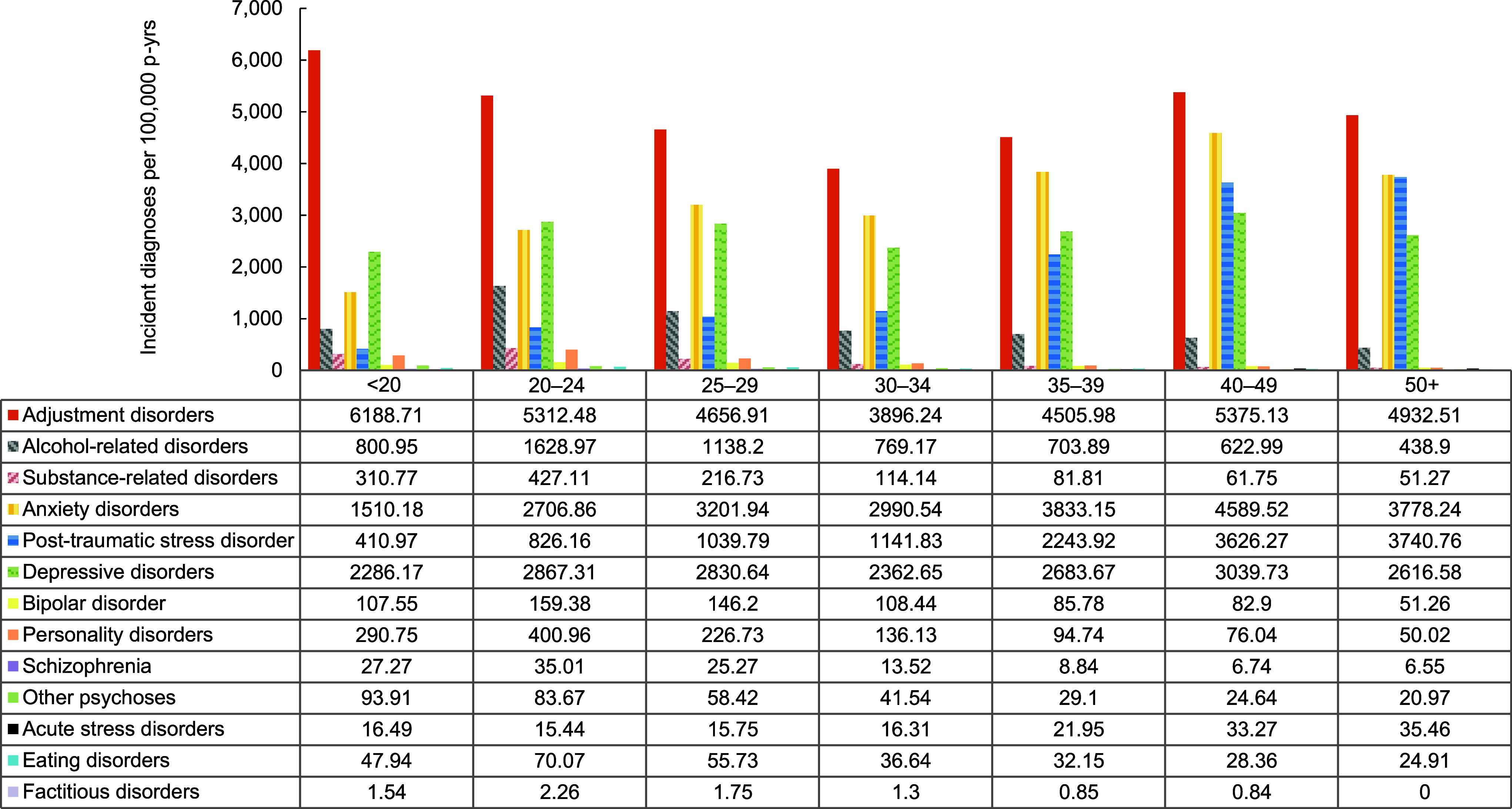
Incidence Rates of Mental Health Disorder Diagnoses, by Age Group and Diagnostic Category, Active Component, U.S. Armed Forces, 2019–2023

**Figure 4 Supplement:**
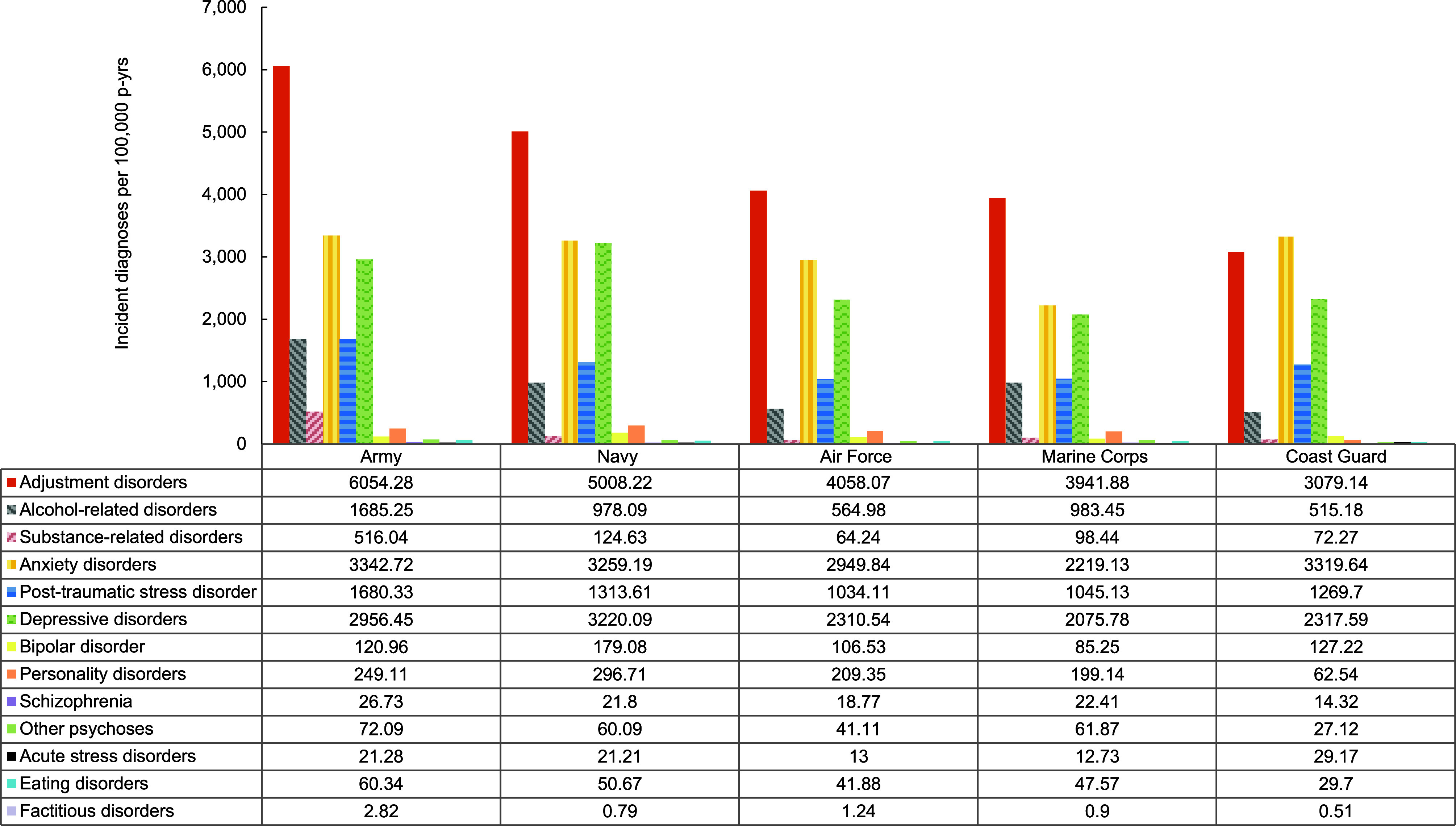
Incidence Rates of Mental Health Disorder Diagnoses, by Service and Diagnostic Category, Active Component, U.S. Armed Forces, 2019–2023

**Figure 5 Supplement:**
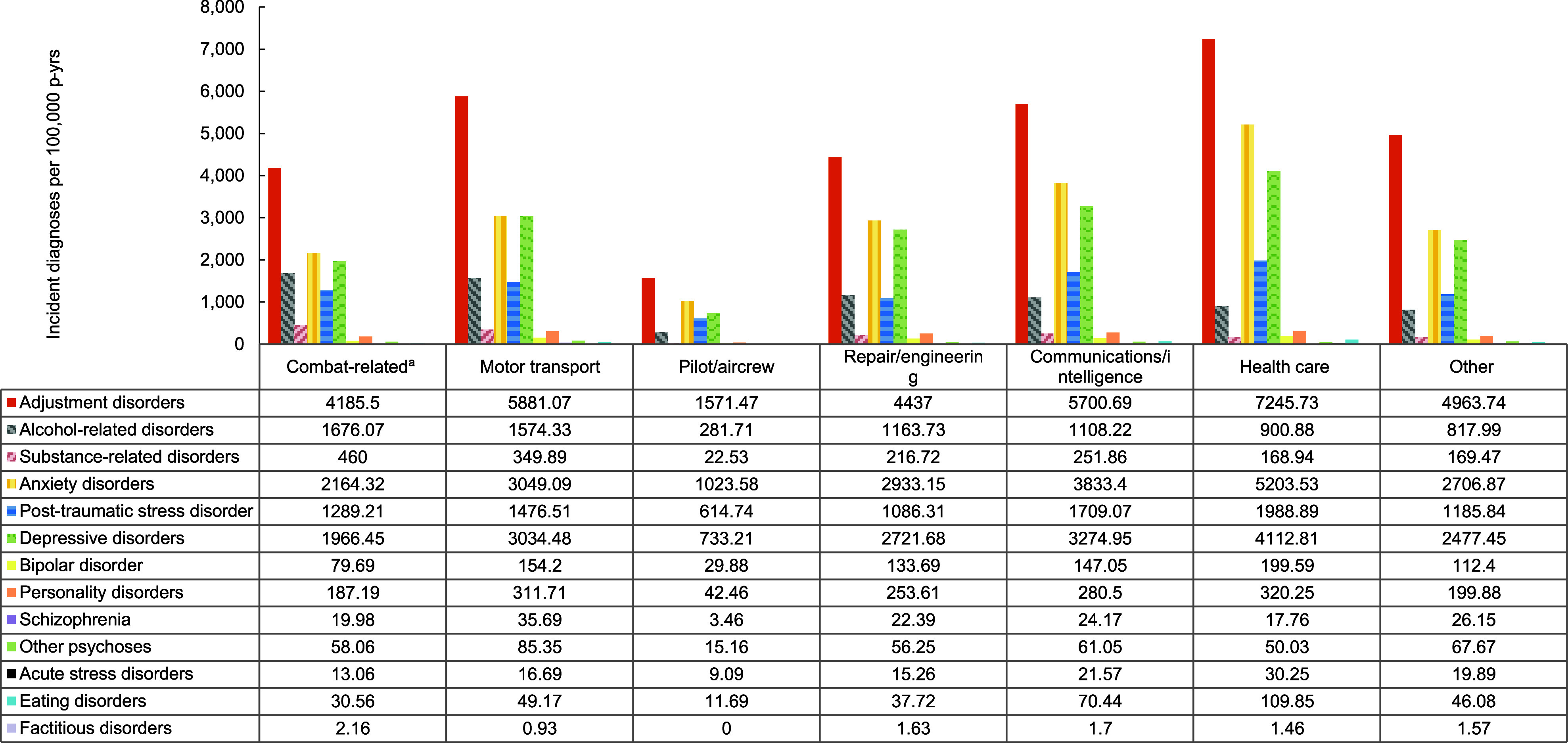
Incidence Rates of Mental Health Disorder Diagnoses, by Military Occupation and Diagnostic Category, Active Component, U.S. Armed Forces, 2019–2023
